# Case Report: Repetitive peripheral magnetic stimulation and task-oriented training improve motor function in chronic severe post-stroke paralysis

**DOI:** 10.3389/fstro.2025.1547280

**Published:** 2025-02-21

**Authors:** Satoshi Yamamoto, Toshiyuki Aoyama, Daisuke Ishii, Kiyoshige Ishibashi, Yutaka Kohno

**Affiliations:** ^1^Department of Physical Therapy, School of Health Sciences, Ibaraki Prefectural University of Health Sciences, Ami, Ibaraki, Japan; ^2^Department of Occupational Therapy, School of Health Sciences, Ibaraki Prefectural University of Health Sciences, Ami, Ibaraki, Japan; ^3^Department of Cognitive Behavioral Physiology, Chiba University Graduate School of Medicine, Chiba, Japan; ^4^Department of Physical Therapy, Ibaraki Prefectural University of Health Sciences Hospital, Ami, Ibaraki, Japan; ^5^Center for Medical Sciences, Ibaraki Prefectural University of Health Sciences, Ami, Ibaraki, Japan

**Keywords:** repetitive peripheral magnetic stimulation, chronic stroke rehabilitation, severe upper-limb paralysis, electromyographic analysis, case study

## Abstract

**Background:**

Severe upper-limb motor paralysis following chronic stroke presents a significant rehabilitation challenge, often with limited recovery. This case study explores the effects of repetitive peripheral magnetic stimulation (rPMS) combined with task-oriented training on motor recovery in a patient with chronic stroke and severe upper-limb impairment.

**Methods:**

A 50-year-old male with right upper-limb paralysis post-hemorrhagic stroke underwent a 2-week intervention comprising 12 sessions of rPMS targeting the elbow and wrist extensors, combined with task-oriented training. Motor function was assessed using the Fugl-Meyer Assessment (FMA), kinematic analysis, Motor Activity Log (MAL), and electromyographic (EMG) analysis of wrist flexion-extension movements.

**Results:**

The intervention resulted in a clinically meaningful increase in motor function, reflected in improved FMA scores and greater elbow extension during kinematic analysis. EMG analysis demonstrated reduced co-contractions of wrist flexors and extensors, indicating improved muscle coordination. Despite these gains, recovery of distal voluntary movements, such as wrist dorsiflexion and finger extension, remained limited. As assessed by MAL, upper-limb usage in daily activities showed minor improvements; however, qualitative reports indicated functional gains, including the ability to hold a bottle and assist in closing a car door.

**Conclusion:**

rPMS combined with task-oriented training shows promise in enhancing motor function in patients with chronic stroke combined with severe upper-limb paralysis, particularly in proximal muscles. Further research involving control groups and objective measures of upper-limb use is necessary to validate these findings and refine intervention protocols.

## 1 Introduction

Post-stroke upper limb paralysis significantly impairs activities of daily living (ADLs), with functional recovery posing a substantial challenge, particularly for patients with moderate to severe motor impairments (Choi, 2022). Rehabilitative strategies such as task-oriented training and repetitive motor practice have demonstrated efficacy for mild to moderate impairments (Pollock et al., 2014; French et al., 2016). However, only 12% of patients achieve full functional recovery, leaving severe cases an ongoing focus in stroke rehabilitation (Aqueveque et al., 2017; Broeks et al., 1999; Kwakkel et al., 2003; Coscia et al., 2019).

Patients with severe upper-limb paralysis often exhibit hypertonia and markedly reduced voluntary motor function, limiting the feasibility of traditional task-oriented training (Pundik et al., 2019). Emerging technologies, such as peripheral nerve electrical stimulation, robotics, and brain-machine interfaces, have shown promise when combined with functional training (Carrico et al., 2016; Bertani et al., 2017; Monge-Pereira et al., 2017; Cervera et al., 2018; Cho et al., 2018; Mehrholz et al., 2018; Carvalho et al., 2019; Conroy et al., 2019; Bai et al., 2020). Despite its reported efficacy, peripheral nerve stimulation is often associated with pain and discomfort (Yoshida et al., 2017; Yang et al., 2019).

Repetitive peripheral magnetic nerve stimulation (rPMS) has emerged as a viable alternative, offering deeper, pain-free stimulation (Beaulieu and Schneider, 2013; Han et al., 2006). Studies indicate that rPMS promotes proprioceptive input and neuroplasticity by inducing a repetitive contraction-relaxation cycle (Struppler et al., 2003; Brown et al., 2009). Clinical benefits have been reported in the acute phase of stroke (Obayashi and Takahashi, 2020; Jiang et al., 2022), and recent findings by Fawaz et al. (2023) suggest motor improvements in patients with chronic-phase stroke following rPMS combined with active training. However, the effects on severe upper limb motor dysfunction remain unclear, as severity-specific analyses were not conducted. To address this gap, our protocol integrates rPMS with task-oriented training, specifically targeting a patient with severe deficits in upper-limb function. Through this approach, we aim to examine whether the combined intervention leads to significant improvements in proximal motor function within this specific patient population.

The severity of upper limb motor paralysis depends on factors such as lesion location and time since stroke onset (Okamoto et al., 2021; Van Der Vliet et al., 2020). Evidence suggests that proximal muscles are easier to target and train than distal ones, particularly in patients with severe impairment (Hijikata et al., 2020; Chen et al., 2023). Tailoring rPMS protocols to the difficulty hierarchy of motor tasks may optimize recovery outcomes for this population.

This case report examined the effects of combining rPMS with task-oriented training in a patient with chronic stroke with severe upper-limb motor paralysis. It aims to provide insights into how targeting proximal muscles based on functional difficulty can contribute to meaningful motor recovery, addressing a key gap in the current literature.

## 2 Material and methods

### 2.1 Experiment design

This study was conducted over 2 weeks, comprising 12 intervention sessions that combined rPMS and task-oriented upper limb training. rPMS was administered before the task-oriented training, and evaluations were performed both before and after intervention. The study received ethical approval from the affiliated institution's ethics committee (approval number e428), and written informed consent was obtained after providing the patient with a detailed explanation of the study procedures.

### 2.2 Patient

**Figure 1:**
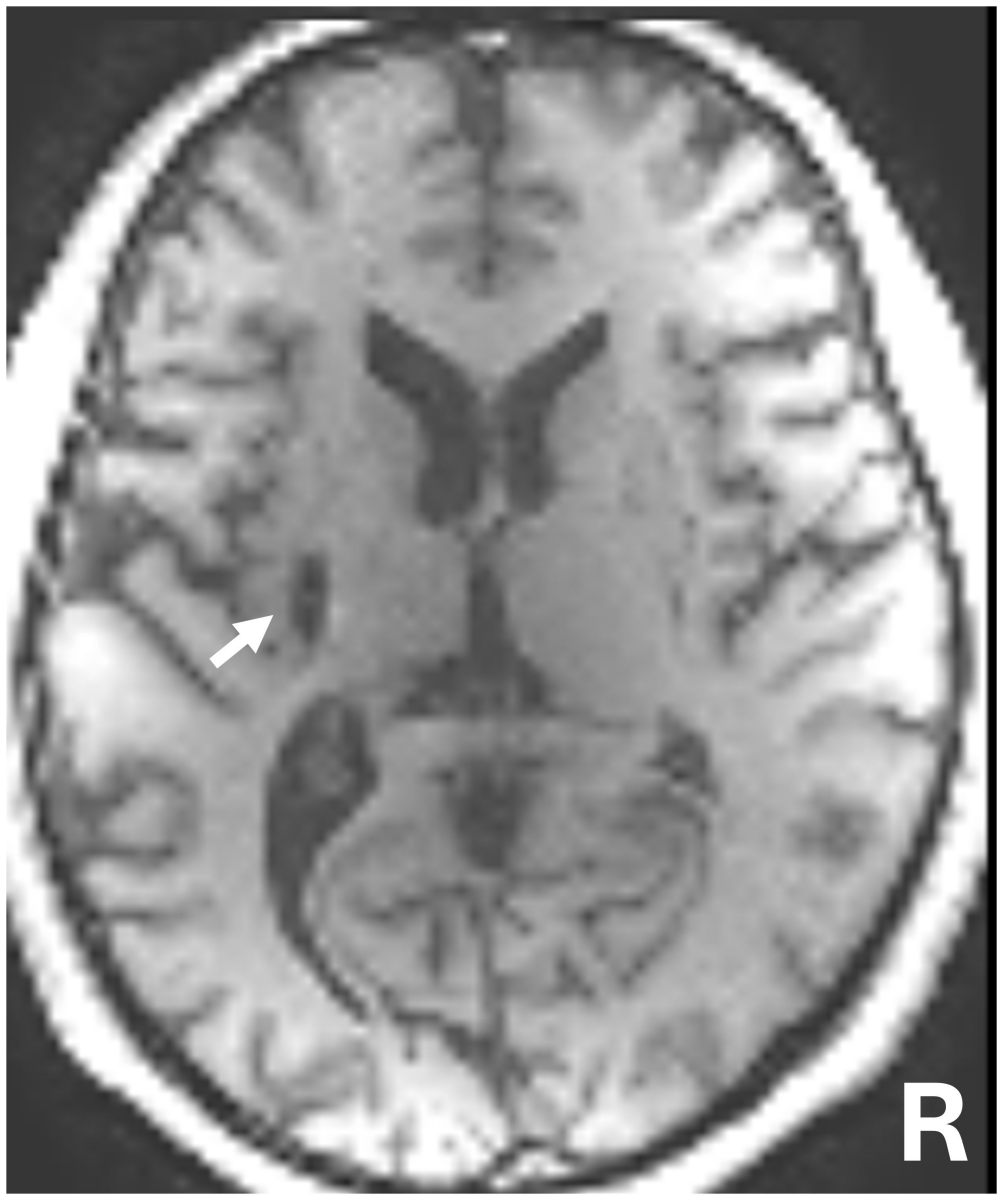
T1-weighted magnetic resonance imaging (MRI) 5 years following brain hemorrhage onset. The white arrow indicates the left putamen lesion.

The patient was a 50-year-old man with chronic right hemiparesis following a left putaminal hemorrhage 5 years prior (Figure 1). The patient initially underwent 5 months of intensive inpatient rehabilitation following stroke onset. Four years post-stroke, he also participated in outpatient physical therapy sessions (once to three times per month), which included task-oriented training. However, no marked improvements in upper-limb function were observed, and these sessions were discontinued 8 months before the start of this study. Since then, the patient has not received any rehabilitation interventions. When the patient began outpatient physical therapy 1 year prior to this study, his shoulder flexion was limited to 150° and abduction to 120°, with no other noted range of joint motion restrictions.

At the beginning of this study, although independently ambulatory and capable of driving, his functional activities were significantly restricted due to limited voluntary use of the affected upper limb. The patient exhibited partial active finger flexion, with no ability to extend the fingers or perform voluntary wrist flexion/extension. Voluntary elbow extension was limited not only by motor paralysis of the elbow extensors but also by increased muscle tone of the elbow flexors (Modified Ashworth Scale score: 2) (Pandyan et al., 1999). The patient's primary therapeutic goals were to improve elbow and wrist movement and enhance functional independence in activities of daily living and occupational tasks.

### 2.3 rPMS

rPMS was administered using a high-frequency peripheral magnetic stimulator (Pathleader, IFG Corporation, Sendai, Japan) (Suzuki et al., 2015). The device generated biphasic pulses (328–372 μs) with a maximum magnetic gradient of 18.2 kT/s. Key stimulation parameters included frequency, 30 Hz (Obayashi and Takahashi, 2020); duty cycle, 2 s ON and 3 s OFF (Fujimura et al., 2020); session duration, 7 min per targeted muscle group; and stimulations per session, ~5,000 pulses (Krewer et al., 2014).

Target muscles included the elbow extensors and wrist dorsiflexors, selected based on a preliminary functional assessment and the patient's rehabilitative goals. Stimulation intensity was adjusted daily to achieve observable joint movement, corresponding to 45%−60% of the device's maximum output.

The procedure identified stimulation sites by eliciting robust muscle contractions in the contralateral limb and subsequently targeting the homologous regions on the paretic limb. rPMS was first applied to the wrist dorsiflexor muscles and then, in a separate session, to the elbow extensor muscles. During the rPMS intervention, the patient was instructed to synchronize voluntary movements with those of the stimulating muscle. However, if the muscle activity of the antagonist muscle inhibited joint movements induced by rPMS, voluntary movements were not synchronized during the rPMS intervention.

### 2.4 Task-oriented training

The task-oriented upper limb training comprised 30-min sessions aimed at progressively challenging motor function through diverse upper-limb exercises. Training components included grasping and releasing objects of varying dimensions, weights, and frictional properties (e.g., spherical balls, cylindrical pegs, and planar boards); table-level tasks, focusing on manipulative skills; and activities requiring elbow extension and wrist dorsiflexion, such as transferring objects beneath the table surface. A custom-fitted finger orthosis was used to prevent hyperflexion of the thumb's interphalangeal joint.

### 2.5 Outcome measures

#### 2.5.1 Motor function and use of the upper limbs in ADLs

Motor function was assessed using the Fugl-Meyer Assessment (FMA) upper limb subscale (Fugl-Meyer et al., 1975). The finger-to-nose test, a subcomponent of the FMA, was video-recorded for kinematic analysis. Two-dimensional trajectory data were collected to measure relative movement between the paralyzed hand and the patient's nose. Standardized reference points (shoulder and hip midpoints) were used, and the maximum distance across five repetitions was calculated.

Motor Activity Log (MAL) (Van Der Lee et al., 2004) outcomes assessed the frequency and quality of upper limb use during functional tasks. On the final day, a semi-structured interview documented qualitative changes in upper limb utilization.

#### 2.5.2 Kinematic and electromyographic analyses

Kinematic and EMG analyses were performed before and after stimulation on both intervention days. The patient was seated and rested the forearm on a table at a height of 70 cm. The shoulder was slightly flexed and internally rotated, the elbow was flexed, the forearm was in a neutral position between pronation and supination (with assistance provided during the measurement), and the wrist was in a neutral position between dorsiflexion and palmar flexion. The tasks included repetitive wrist palmar/dorsal flexion and finger flexion/extension (performed at a controlled cadence of one cycle every 6 seconds under verbal guidance). Video recordings were acquired from an overhead perspective to ensure clear visualization of the forearm and hand. The Supplementary videos 1, 2 (1: Finger Flexion-Extension Task, 2: Wrist Flexion-Extension Task) present synchronized recordings of the EMG signals and the patient's movement.

For kinematic analysis, joint angles (elbow, wrist, metacarpophalangeal [MP] joint, and fingertip of the index finger) were calculated using Kinovea software (2023.1). This method was adapted from a previous study (Aoyama et al., 2021), in which markers were placed on the patient's joints, and a two-dimensional motion analysis using a video camera was employed to calculate joint angles. Data smoothing was achieved using a 10 Hz second-order Butterworth low-pass filter and a 1-s moving average.

All kinematic and EMG data were processed using MATLAB R2024a (MathWorks, Natick, USA). The wrist extension angle was defined as the angle subtended by the elbow, wrist, and MP joint, while the finger flexion angle was measured as the angle formed by the wrist, MP joint, and fingertip. A 10 Hz second-order Butterworth low-pass filter was applied to smooth the data, followed by a 1-s moving average.

EMG signals were recorded using a wireless system (TS-MYO, Trunk Solution, Japan) at 1 kHz sampling. Bipolar surface electrodes targeted the flexor and extensor carpi radialis muscles. Signal processing involved detrending, high-pass filtering (10 Hz Butterworth), rectification, and smoothing. EMG amplitudes were normalized to a maximum value of 100% for inter-condition comparisons.

## 3 Results

### 3.1 Motor paralysis and use of the upper limbs in ADLs

The FMA score was improved from 27 at baseline to 33 post-intervention (Supplementary Table 1), surpassing the threshold for minimal detectable change (MDC). Improvements were observed in shoulder, elbow, and forearm flexion joint movement (+3 points) and hand grip movement (+3 points).

**Figure 2:**
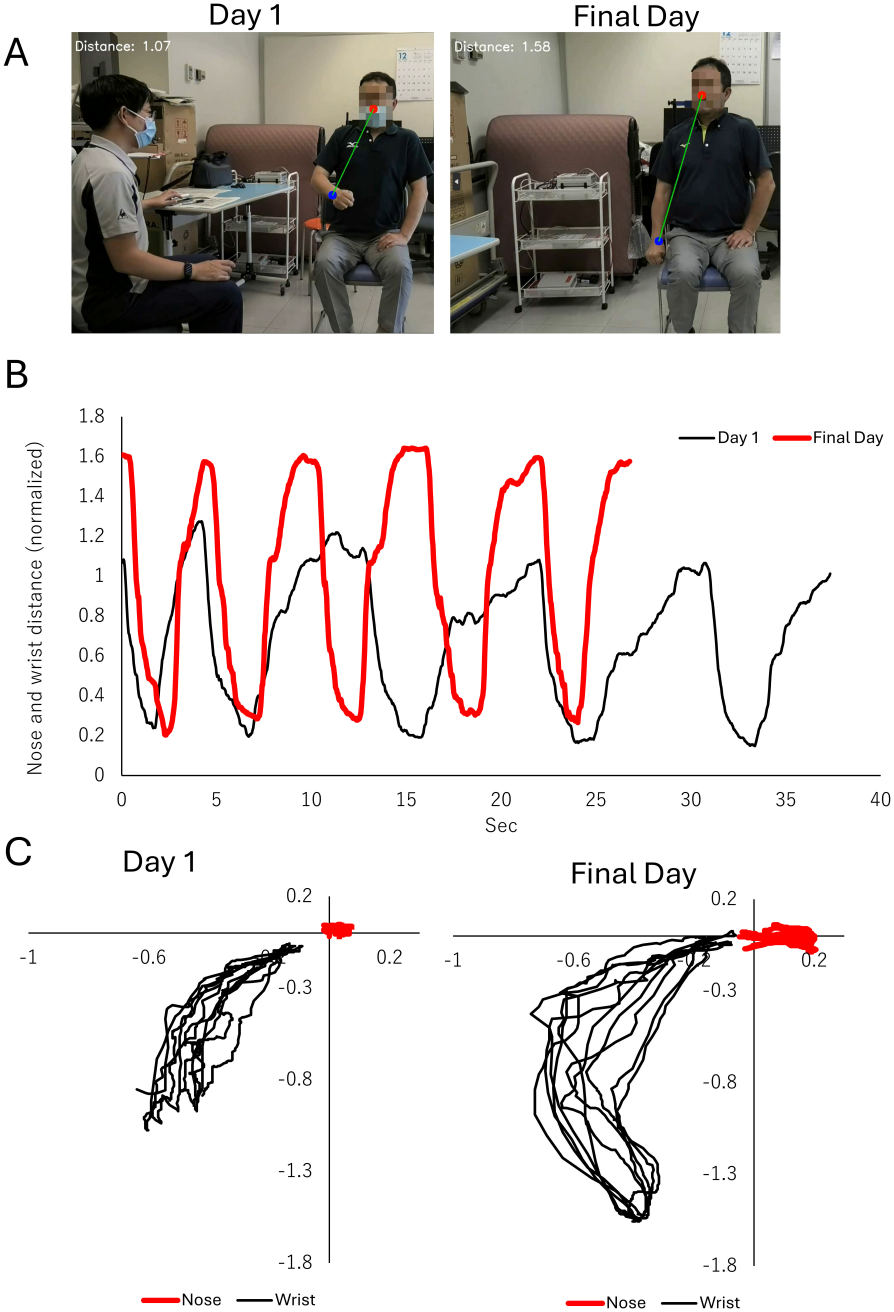
Improvements in the finger-to-nose test, a subcomponent of the fugl-meyer assessment (FMA). **(A)** Representative images showing the maximum distance (green line) between the nose (red circle) and the wrist of the paralyzed side (blue circle). The left panel illustrates performance on Day 1 of the intervention, while the right panel shows performance on the final day of the intervention. **(B)** Time-series plot of the distance between the nose and wrist of the paralyzed side during the finger-to-nose task. The black line represents Day 1, and the red line represents the final day of intervention. Improvements in distance and task performance are evident. **(C)** Two-dimensional trajectories of the nose (red line) and wrist (black line) of the paralyzed side in the frontal plane. The left panel displays trajectories on Day 1, characterized by restricted and inconsistent wrist movement. The right panel depicts trajectories on the final day, showing a broader range of wrist movement and improved task execution.

The finger-nose test score remained at 2 points, but task performance improved: completion time reduced from 38 s to 27 s (Figure 2B), and the range of motion between the nose and the paralyzed hand increased considerably (from 1.13 ± 0.11 meters to 1.60 ± 0.03) (Figures 2A–C).

MAL outcomes included amount of use (AOU), 1.79 ± 2.49 pre-intervention to 1.86 ± 2.44 post-intervention. Quality of movement (QOM) also improved from a baseline of 0.71 ± 1.27 to 0.93 ± 1.38 on the last day.

For the sub-items, the QOM for “putting one's hand through the sleeve of a garment” increased from 2 to 3 points. For “moving an object with one's hand,” AOU and QOM improved, with AOU increasing from 0 to 1 point and QOM increasing from 0 to 2 points. During the patient interview, it was noted that on the final day of the intervention, the patient was able to hold a plastic bottle with the paralyzed hand while opening it, an action that was not previously possible. Additionally, the patient, who had been unable to use the paralyzed hand to close a car door prior to the intervention, was now able to position the paralyzed hand using the non-paralyzed hand and successfully pull the door closed with the paralyzed hand.

### 3.2 Kinematic and EMG evaluation of palmar and dorsal flexion movements

**Figure 3:**
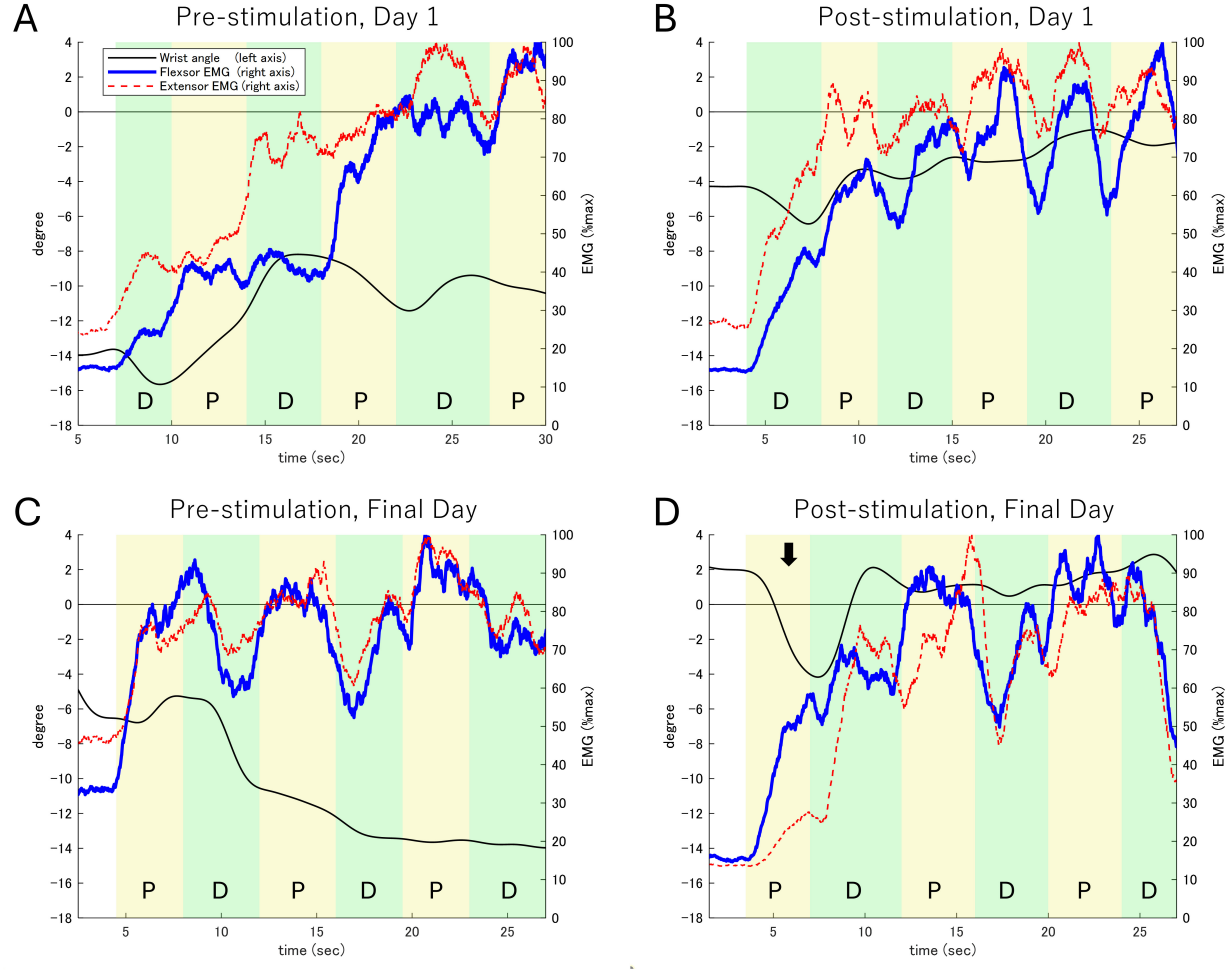
Electromyographic (EMG) activity and wrist extension angle during the wrist flexion-extension task. **(A)** Pre-stimulation on Day 1. **(B)** Post-stimulation on Day 1. **(C)** Pre-stimulation on the Final Day. **(D)** Post-stimulation on the Final Day. The left Y-axis represents the wrist extension angle (black line), while the right Y-axis shows the EMG activity of the wrist flexor (blue line) and extensor (red dashed line) muscles. The task consists of alternating phases: Dorsiflexion (D) and Palmar flexion (P). The black arrow in **(D)** highlights the first palmar flexion phase on the Final Day (Post-stimulation), where a notable increase in palmar flexion movement and corresponding activity of the palmar flexor muscles is observed. In contrast, the activity of the dorsal flexor muscles shows limited increases, reflecting improved motor control during the task.

In the wrist palmar flexion-extension task (Figure 3), pre-stimulation results on Day 1 revealed a flexion angle of −14° and a limited dorsiflexion range (from −16° to −8°) due to persistent antagonist muscle contraction.

Post-stimulation observations included:

Day 1: The dorsiflexion angle improved to −4°, though antagonist contraction persisted, limiting further gains.

Final Day (pre-stimulation): Dorsiflexion began at −5°, gradually improving to −14° over task execution.

Final Day (post-stimulation): The dorsiflexion angle improved to 2°, with increased dorsal flexor activity contributing to improved movement control. Fluctuations between −4° and 2° were observed, with more time spent in a dorsiflexed position than baseline.

These findings suggest enhanced motor coordination and reduced antagonist co-contraction following repeated rPMS sessions. Supplementary EMG waveforms (Supplementary Figure 1) demonstrate increased activation of dorsal flexors and reduced compensatory activity in palmar flexors.

After stimulation on the first day (Figure 3B), the dorsiflexion angle improved to −4°. However, simultaneous contraction of the flexor and extensor muscles limited changes in the dorsiflexion angle to within 2° during each phase. On the final day before stimulation (Figure 3C), the dorsiflexion angle was −5°, with continued simultaneous muscle contraction gradually reducing the dorsiflexion range to −14° over the task duration.

On the final day after stimulation (Figure 3D), the dorsiflexion angle improved to 2°, accompanied by marked palmar flexor activity, resulting in a 6° palmar flexion movement. In subsequent phases, dorsal flexor activity increased, producing approximately 6° of dorsiflexion, although palmar flexor activity persisted. Throughout the task, the dorsiflexion angle fluctuated between −4° and 2°, with increased time spent in the dorsiflexion position.

Additional EMG waveforms and detailed analyses are available as Supplementary Figure 1.

### 3.3 Kinematic and EMG evaluation of finger flexion and extension tasks

During the finger flexion-extension task (Supplementary Figures 2, 3), we did not observe any increase in extensor muscle activity during the extension phase (when flexor muscles were inactive) on Day 1 or before stimulation on the final day. However, in the second extension phase following stimulation, there was a slight increase in extensor muscle activity, while the flexor muscles did not show increased activity.

### 3.4 Adverse effects

No adverse events, such as pain, discomfort, or unexpected reactions, were reported during the intervention. All sessions were completed as planned, confirming the safety and tolerability of the combined rPMS and task-oriented training protocol.

## 4 Discussion

This study investigated the effects of combining rPMS and task-oriented training in a patient with severe upper motor paralysis combined with chronic-phase stroke. The intervention resulted in a 6-point improvement in FMA, reduced time required for the finger-nose test, increased upper limb range of motion, and a change in muscle activity during palmar flexion. These improvements exceed the MDC (3.2–7.2) and minimally clinically important difference (MCID; 4.0–12.4) thresholds reported for FMA in upper-limb stroke rehabilitation (Hsueh et al., 2008; Lin et al., 2009; See et al., 2013; Arya et al., 2011; Page et al., 2012; Lundquist and Maribo, 2017; Hiragami et al., 2019). The findings suggest that rPMS combined with task-oriented training has clinical relevance in promoting motor recovery even in the chronic phase.

The patient exhibited severe motor paralysis at baseline, with an FMA score below 30 points and the inability to perform voluntary finger and wrist extension. These findings align with prior definitions of severe motor impairment, including a total FMA score of ≤ 30 (Coscia et al., 2019) and limited extension of the metacarpophalangeal and wrist joints (Carrico et al., 2016). Targeting the elbow and wrist extensors with rPMS led to noticeable improvements in elbow extension range and reductions in antagonist muscle activity, particularly in the extensor carpi radialis, during palmar flexion. However, voluntary distal motor movements, including finger extension or wrist dorsiflexion, showed limited improvement.

The limited improvement in distal motor control is consistent with the findings of Hijikata et al. (2020), who reported that task difficulty in FMA increases from proximal to distal muscles, with tasks involving the hand being the most challenging. Additionally, antagonist overactivity is a well-known factor interfering with voluntary movement in patients with motor paralysis (Pundik et al., 2019). In this study, the reduction in antagonist activity likely facilitated proximal motor gains, suggesting that appropriate targeting of muscles for rPMS can promote task-specific improvements. However, recovery of distal muscle function remains a challenge in severe cases and may require additional interventions or longer treatment durations.

Previous studies examining rPMS and upper limb training often lacked detailed descriptions of motor tasks, focusing primarily on passive stretching or automated joint range of motion exercises (Obayashi and Takahashi, 2020; Werner et al., 2016; Krewer et al., 2014). In contrast, this study incorporated task-oriented training, which may enhance neural circuit activity and promote motor learning. Combining rPMS with functional, task-oriented practice likely facilitated motor recovery that simple upper-limb exercises alone could not achieve. However, further research is needed to verify these findings, particularly in patients with severe paralysis in chronic-phase stroke.

In this study, although MAL was used to assess upper limb use, the results did not meet the MDC thresholds for patients with stroke: 0.84 for AOU and 0.77 for QOM (Chen et al., 2012). The MCID for dominant-hand paralysis is reported as 1.0 (Lang et al., 2008). Despite this, notable qualitative improvements were observed, such as elbow extension and specific upper limb tasks (e.g., manipulating objects) that were previously unachievable. These findings are further supported by changes in FMA scores, kinematic evaluations, and EMG data. While the FMA evaluates motor function through standardized assessment tasks, the MAL reflects the patient's ability to use their affected limb in everyday activities. This difference in focus may explain the discrepancies in outcomes.

This study has some limitations. First, the absence of a control group makes it challenging to attribute the observed motor improvements solely to the intervention. However, as the patient was in the chronic phase of stroke recovery, significant natural recovery in upper limb motor function is unlikely (Hussain et al., 2021; Borschmann and Hayward, 2020).

Second, while the intervention required only 1 hour per day over 14 days, the patient was encouraged to practice independently and integrate the acquired functions into daily activities and work. The actual usage of the affected upper limb likely exceeded the intervention time. However, the lack of objective measurement tools, such as activity monitors, limits our ability to quantify the real-world use of the upper limb.

Finally, this intervention requires substantial voluntary effort from patients, which may restrict its applicability to individuals with cognitive or motivational limitations.

Although these findings are promising, future research should include control groups and incorporate objective evaluations of upper limb usage to comprehensively validate the intervention's effectiveness.

## 5 Conclusion

In this study, we investigated the effects of rPMS combined with task-oriented training in a patient with severe upper limb motor paralysis in the chronic phase after a brain hemorrhage. The interventional resulted in clinically meaningful improvements, including increased FMA scores and enhanced range of motion, particularly in proximal muscle groups.

However, improvements in voluntary movements of distal muscles, which are inherently more challenging to recover, were limited. Additionally, there was no significant change in the amount or quality of upper limb usage during daily activities.

Future research should include control groups to validate these findings and employ objective measures such as activity monitors to accurately assess upper limb use in real-world settings. This will further clarify the intervention's efficacy and guide the development of personalized rehabilitation protocols.

## Data Availability

The original contributions presented in the study are included in the article/Supplementary material, further inquiries can be directed to the corresponding author.
