# Supplementary material for: Case Report: Repetitive peripheral magnetic stimulation and task-oriented training improve motor function in chronic severe post-stroke paralysis
Source: Front Stroke. 2025 Feb 21;4:1547280. doi: 10.3389/fstro.2025.1547280 (PMC12802746; doi:10.3389/fstro.2025.1547280)
Supplement: Supplementary file 3 [file Data_Sheet_1.docx]

Supplementary Material

# Supplementary Figures


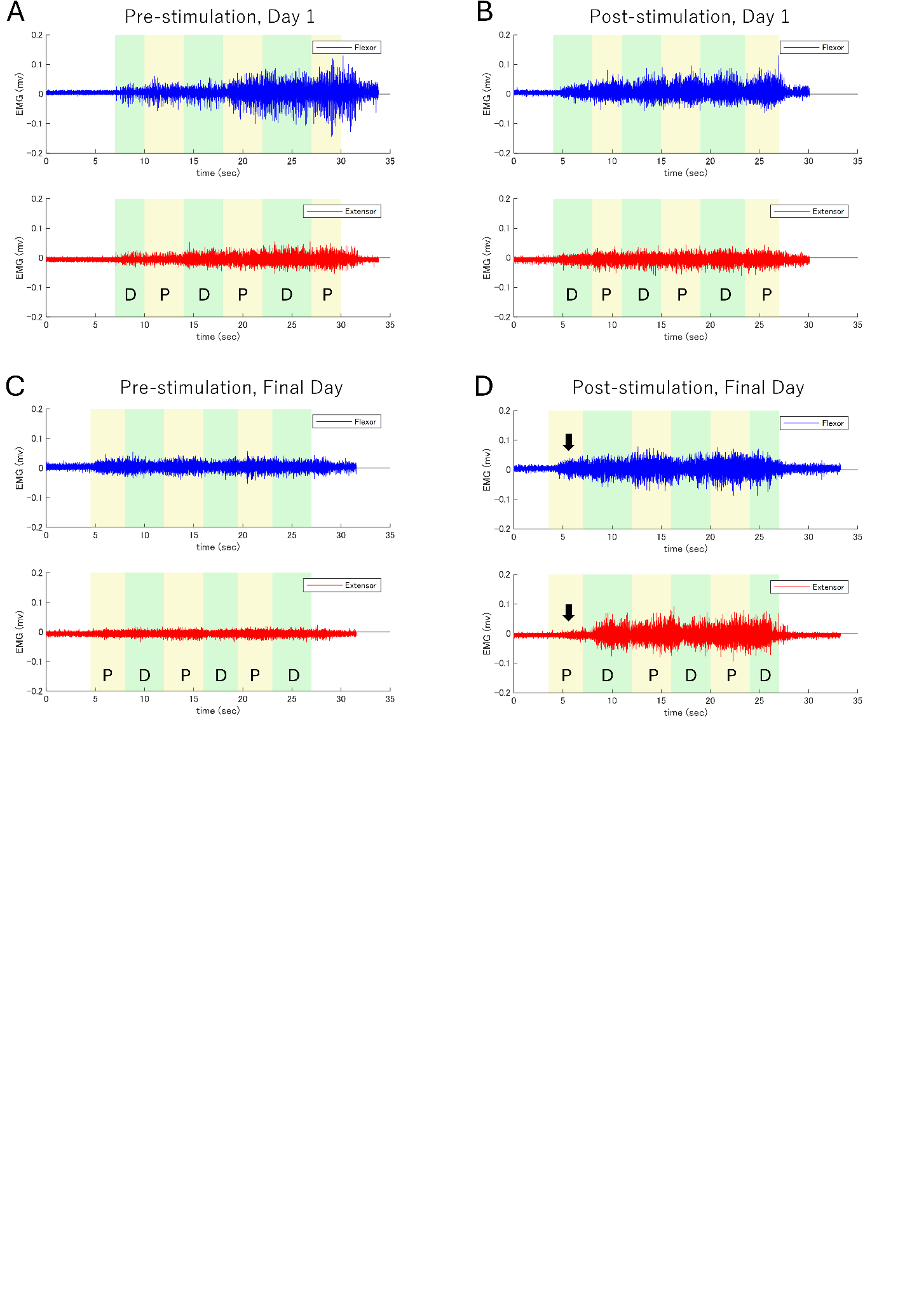


**Supplementary Figure 1.** Raw Electromyographic (EMG) Waveforms During the Wrist Flexion-Extension Task.

(A) Pre-stimulation on Day 1. (B) Post-stimulation on Day 1. (C) Pre-stimulation on the Final Day. (D) Post-stimulation on the Final Day. The blue line represents the EMG activity of the wrist flexor muscles, and the red line represents the EMG activity of the wrist extensor muscles. The alternating phases of the wrist movement task are labeled as D: Dorsiflexion phase; P: Palmar flexion phase. In panel D (Post-stimulation, Final Day), the black arrows highlight the first palmar flexion phase, where increased palmar flexor activity is observed alongside limited activity in the dorsal flexor muscles, indicating reduced co-contraction and improved motor control.


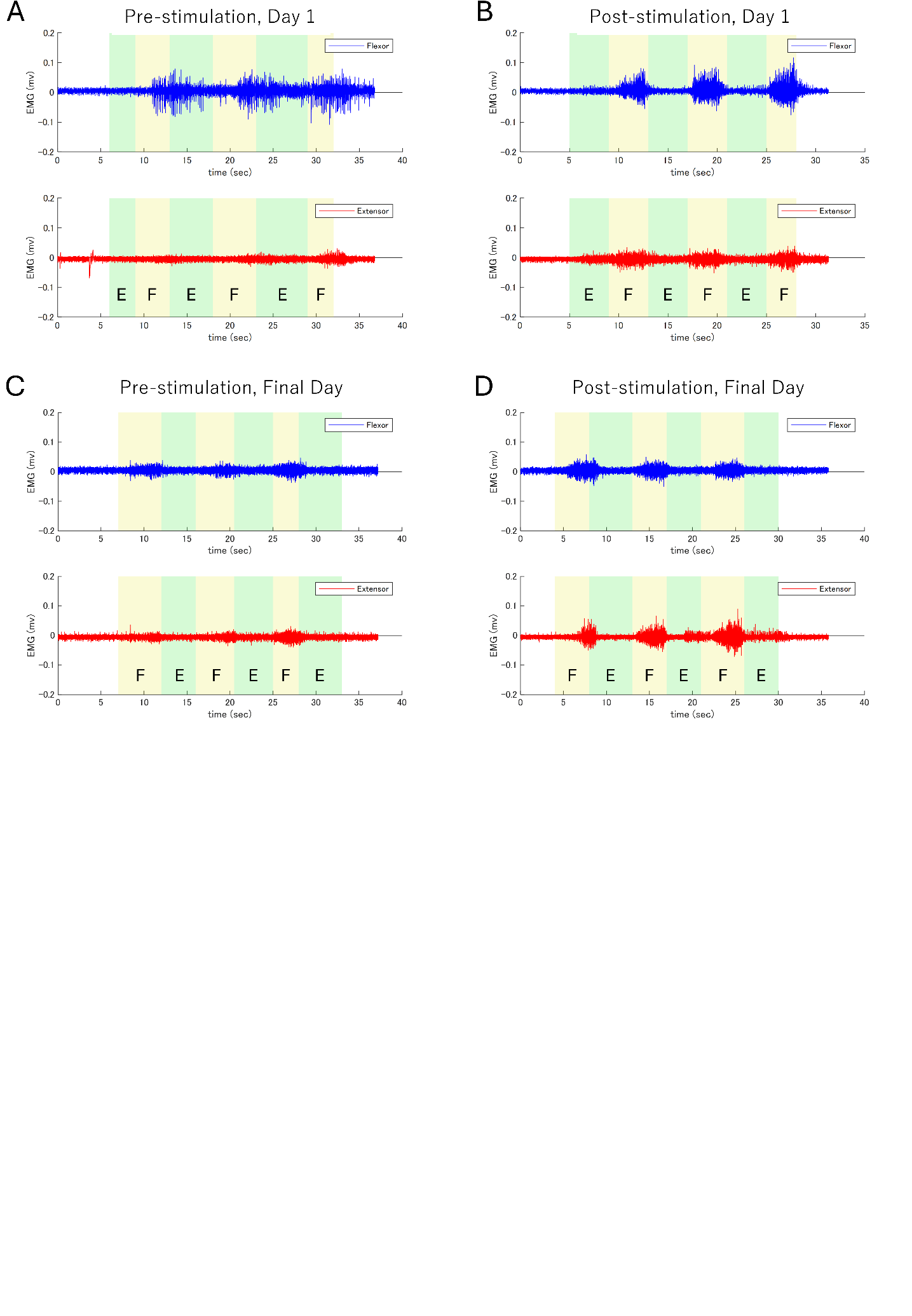


**Supplementary Figure 2.** Raw Electromyographic (EMG) Waveforms During the Finger Flexion-Extension Task.

(A) Pre-stimulation on Day 1. (B) Post-stimulation on Day 1. (C) Pre-stimulation on the Final Day. (D) Post-stimulation on the Final Day. The blue line represents the EMG activity of the finger flexor muscles, while the red line represents the EMG activity of the finger extensor muscles. Alternating phases of the task are labeled as follows: E: Finger extension phase; P: Finger flexion phase. In panel D (Post-stimulation, Final Day), increased flexor activity is observed during the finger flexion phase (F), while extensor activity during the finger extension phase (E) remains relatively low. This suggests improved motor selectivity and reduced co-contraction of antagonist muscles following the intervention.


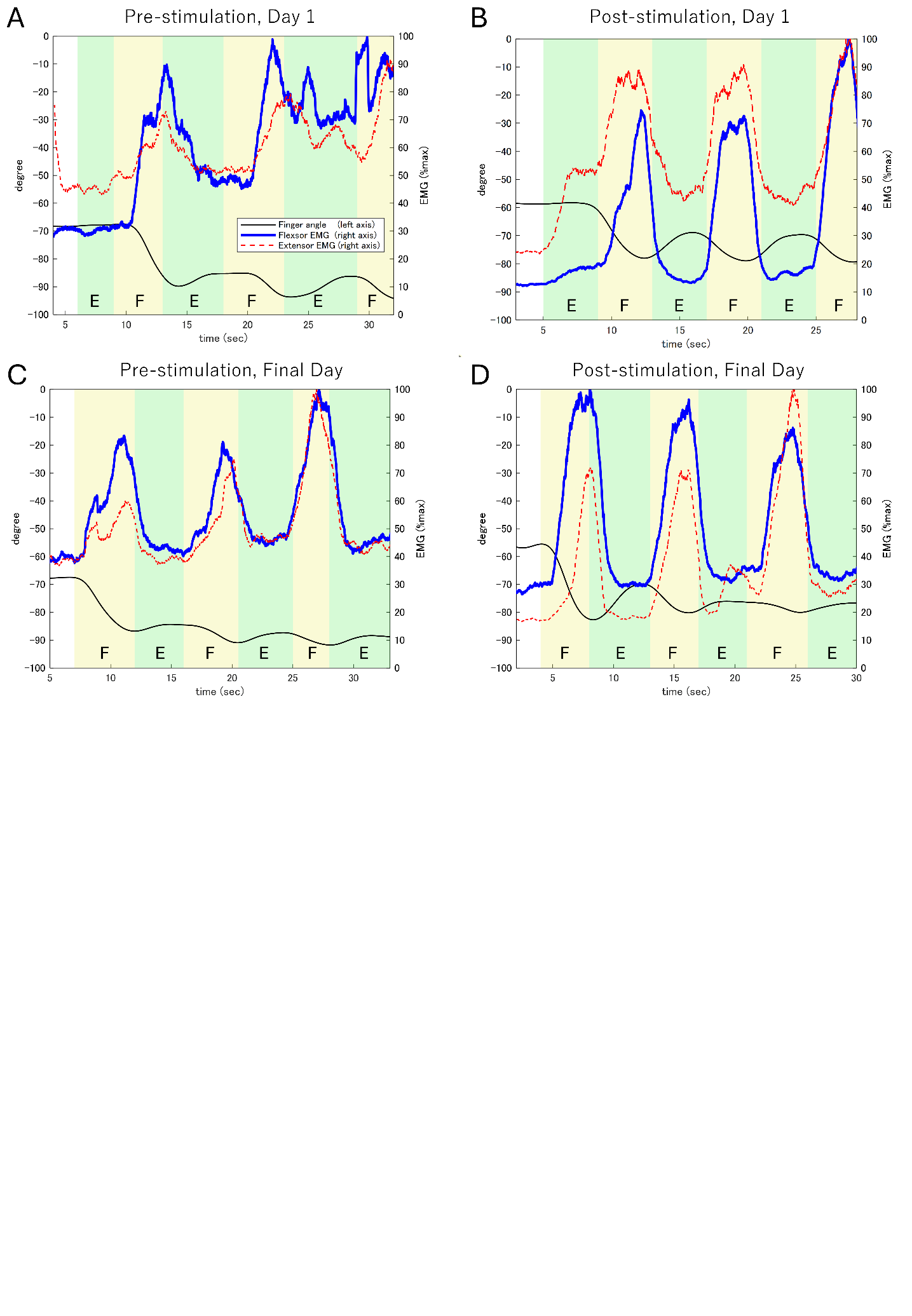


**Supplementary Figure 3.** Electromyographic (EMG) and Finger Flexion Angle During the Finger Flexion-Extension Task.

(A) Pre-stimulation on Day 1. (B) Post-stimulation on Day 1. (C) Pre-stimulation on the Final Day. (D) Post-stimulation on the Final Day. The left Y-axis represents the finger flexion angle (black line), while the right Y-axis shows the EMG activity of the finger flexor (blue line) and extensor (red dashed line) muscles. The task alternates between: E: Finger extension phase; P: Finger flexion phase. In panel D (Post-stimulation, Final Day), improvements are observed as increased finger flexion angles coincide with elevated flexor muscle activity (blue line). Notably, extensor muscles (red dashed line), which had not previously activated during the extension phase, showed slight activation during the second extension phase, while flexor muscle activity remained unchanged, suggesting improved muscle selectivity and reduced co-contraction.

# Supplementary Table

**Supplementary Table 1.** Fugl-Meyer Assessment of upper extremity.

|  |  |  |  | Pre-stimulation, Day 1 | Post-stimulation, Final Day |
| --- | --- | --- | --- | --- | --- |
| A | I. | Reflex activity Flexors | | 2 | 2 |
| Shoulder |  | Extensors | | 2 | 2 |
| / Elbow | II. | a. Flexor | Shoulder retraction | 1 | 2 |
| / Forearm |  | synergy | Elevation | 2 | 2 |
|  |  |  | Abduction | 2 | 2 |
|  |  |  | External rotation | 1 | 2 |
|  |  |  | Elbow flexion | 1 | 2 |
|  |  |  | Forearm supination | 0 | 0 |
|  |  | b. Extensor | Shoulder add/Int. rotation | 2 | 2 |
|  |  | synergy | Elbow extension | 1 | 1 |
|  |  |  | Forearm pronation | 2 | 2 |
|  | III. | Hand to lumber spine | | 0 | 0 |
|  |  | Shoulder flexion 0°-90° | | 1 | 1 |
|  |  | Elbow 90° pro/supination | | 0 | 0 |
|  | IV. | Shoulder abduction 0°-90° | | 1 | 1 |
|  |  | Shoulder flexion 90°-180° | | 1 | 1 |
|  |  | Elbow 0° Pro/supination | | 0 | 0 |
|  | V. | Normal reflex-activity | | 0 | 0 |
| B | Elbow 90° wrist-stability | | | 0 | 0 |
| Wrist | Elbow 90° wrist-flexion/extension | | | 0 | 0 |
|  | Elbow 0° wrist-stability | | | 0 | 0 |
|  | Elbow 0° wrist-flexion/extension | | | 0 | 0 |
|  | Circumduction | | | 0 | 0 |
| C | Fingers mass flexion | | | 2 | 2 |
| Hand | Fingers mass extension | | | 0 | 0 |
|  | Grasp | a. |  | 1 | 2 |
|  |  | b. |  | 0 | 1 |
|  |  | c. |  | 1 | 1 |
|  |  | d. |  | 1 | 2 |
|  |  | e. |  | 1 | 1 |
| D |  | Tremor |  | 1 | 1 |
| Coordination |  | Dysmetria |  | 1 | 1 |
| / Speed |  | Time |  | 0 | 0 |
|  |  |  | Total | 27 | 33 |
